# RNA Sequencing Elucidates Drug-Specific Mechanisms of Antibiotic Tolerance and Resistance in Mycobacterium abscessus

**DOI:** 10.1128/AAC.01509-21

**Published:** 2022-01-18

**Authors:** Jodie A. Schildkraut, Jordy P. M. Coolen, Sophie Burbaud, Jasper J. N. Sangen, Michael P. Kwint, R. Andres Floto, Huub J. M. op den Camp, Lindsey H. M. te Brake, Heiman F. L. Wertheim, Kornelia Neveling, Wouter Hoefsloot, Jakko van Ingen

**Affiliations:** a Radboudumc Center for Infectious Diseases, Department of Medical Microbiology, Radboud University Medical Center, Nijmegen, the Netherlands; b University of Cambridge Molecular Immunity Unit, Department of Medicine, Cambridge, United Kingdom; c Department of Human Genetics, Radboud University Medical Center, Nijmegen, the Netherlands; d Department of Microbiology, IWWR, Radboud University, Nijmegen, the Netherlands; e Radboudumc Center for Infectious Diseases, Department of Pharmacy, Radboud University Medical Center, Nijmegen, the Netherlands; f Radboudumc Center for Infectious Diseases, Department of Pulmonary diseases, Radboud University Medical Center, Nijmegen, the Netherlands

**Keywords:** *M. abscessus*, RNA sequencing, antibiotic resistance, nontuberculous mycobacteria

## Abstract

Mycobacterium abscessus is an opportunistic pathogen notorious for its resistance to most classes of antibiotics and low cure rates. M. abscessus carries an array of mostly unexplored defense mechanisms. A deeper understanding of antibiotic resistance and tolerance mechanisms is pivotal in development of targeted therapeutic regimens. We provide the first description of all major transcriptional mechanisms of tolerance to all antibiotics recommended in current guidelines, using RNA sequencing-guided experiments. M. abscessus ATCC 19977 bacteria were subjected to subinhibitory concentrations of clarithromycin (CLR), amikacin (AMK), tigecycline (TIG), cefoxitin (FOX), and clofazimine (CFZ) for 4 and 24 h, followed by RNA sequencing. To confirm key mechanisms of tolerance suggested by transcriptomic responses, we performed time-kill kinetic analysis using bacteria after preexposure to CLR, AMK, or TIG for 24 h and constructed isogenic knockout and knockdown strains. To assess strain specificity, pan-genome analysis of 35 strains from all three subspecies was performed. Mycobacterium abscessus shows both drug-specific and common transcriptomic responses to antibiotic exposure. Ribosome-targeting antibiotics CLR, AMK, and TIG elicit a common response characterized by upregulation of ribosome structural genes, the *WhiB7* regulon and transferases, accompanied by downregulation of respiration through *NuoA-N.* Exposure to any of these drugs decreases susceptibility to ribosome-targeting drugs from multiple classes. The cytochrome bd-type quinol oxidase contributes to CFZ tolerance in M. abscessus, and the sigma factor *sigH* but not antisigma factor MAB_3542c is involved in TIG resistance. The observed transcriptomic responses are not strain-specific, as all genes involved in tolerance, except *erm(41)*, are found in all included strains.

## INTRODUCTION

Mycobacterium abscessus is a nontuberculous mycobacterium (NTM), capable of causing opportunistic pulmonary infections in patients with local or systemically decreased immunity (1). It is recognized as an important emerging health threat, particularly for susceptible individuals such as cystic fibrosis (CF) patients. Recently it was shown that M. abscessus, unlike other NTM, is human transmissible and a global spread of clustered isolates is seen in CF patients. In addition, the isolates found within these outbreak clusters show increased virulence in mice, likely due to multiple rounds of genetic selection within patients (2, 3).

For its intrinsic resistance to most classes of antibiotics, M. abscessus has rightfully been labeled an “antibiotic nightmare” (4). The major determinant of the intrinsic antibiotic-resistance of M. abscessus is the highly impermeable cell membrane, rendering it resistant to most broad-spectrum antibiotics (5). In addition, M. abscessus uses a range of drug- and target modulating mechanisms and efflux pumps by which resistance to antibiotics is achieved. Examples are the erythromycin resistance methylase (erm) responsible for macrolide resistance, aminoglycoside N-acetyltransferases aac(‘2) and Eis2, the class A β-lactamase Bla_Mab, the MmpL5/MmpS5 bedaquiline and clofazimine (CFZ) efflux system, and the tetracycline-inactivating monooxygenase MabTetX (6–10). As a result, treatment is complex and highly toxic, comprised of at least three drugs with proven *in vitro* activity in the first phase. Regimens typically include intravenous amikacin (AMK), tigecycline (TIG), imipenem or cefoxitin (FOX), with oral clofazimine (CFZ) and a macrolide (preferably azithromycin) for 3 months (11, 12). This is followed by an oral and inhaled continuation regimen, partly based on *in vitro* susceptibilities. Despite this intensive regimen, treatment is only successful in approximately 45% of all patients (13).

Although some mechanisms contributing to the high level of antibiotic resistance in M. abscessus have been elucidated, most remain unknown. To improve treatment and outcome of M. abscessus disease, it is important to understand the mechanisms by which antibiotic tolerance and resistance are acquired. Therefore, we studied the full transcriptomic responses of M. abscessus to all drugs included in the recommended initial treatment regimen. To this end, we exposed M. abscessus to subinhibitory concentrations of each drug and used RNA sequencing to characterize pathways contributing to antibiotic tolerance and confirmed findings in secondary experiments.

## RESULTS

### M. abscessus shows a tailored transcriptomic response to each antibiotic.

**FIG 1:**
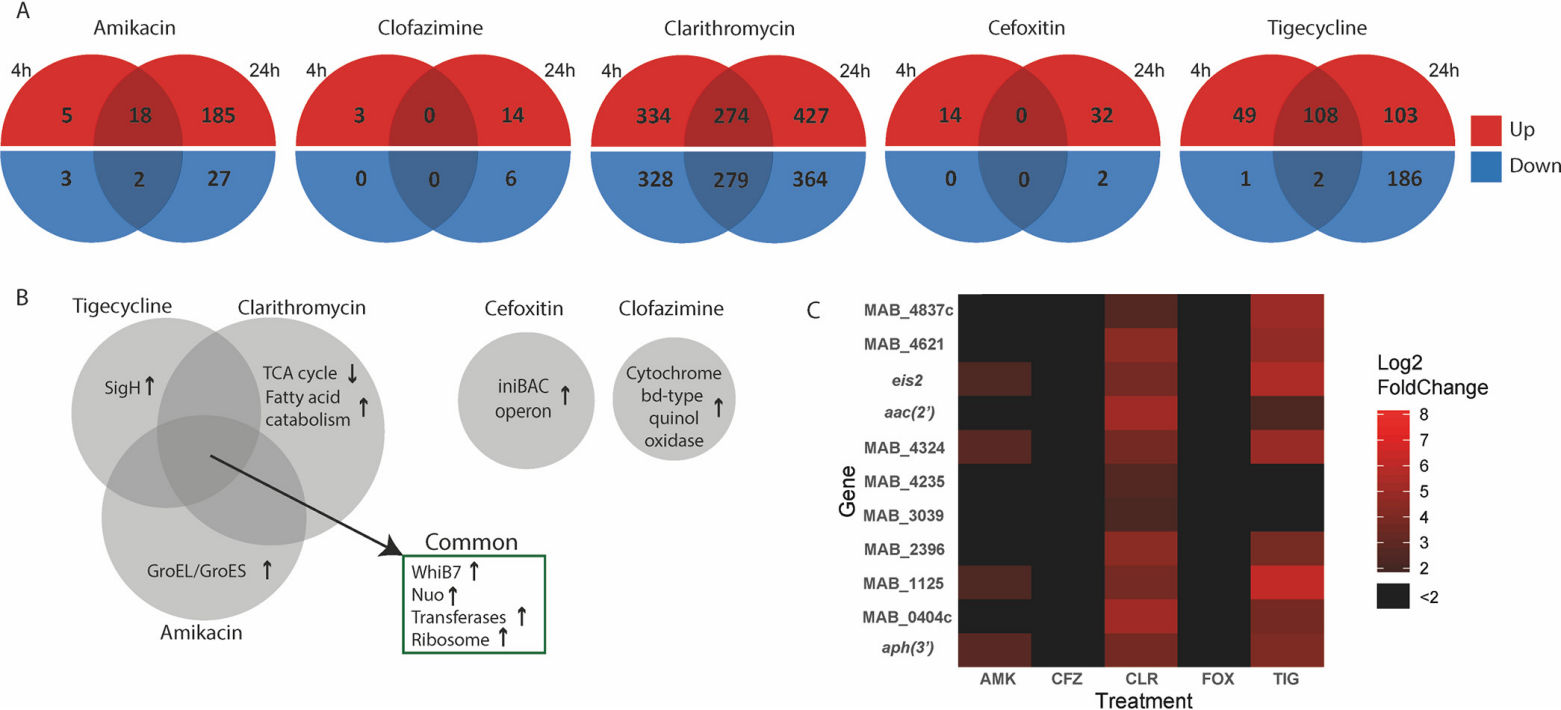
(A) Number of DEGs per condition. (B) Common and condition-specific transcriptomic changes. (C) Differentially expressed transferases. Heatmap illustrating transferases that had a fold change of >2 Log_2_ and a *P* value of <0.05. AMK, amikacin; CFZ, clofazimine; CLR, clarithromycin; FOX, cefoxitin; TIG, tigecycline.

Principal-component analysis (PCA) showed that the antibiotic stress response to each drug was reproducible and drug-specific; PCA plots are shown in Fig. S1 in the supplemental material. The number of differentially expressed genes (DEGs) for each condition and the overlap between responses after 4 and 24 h of exposure are presented in Fig. 1A.

### The transcriptomic response to ribosome-targeting drugs.

**FIG 2:**
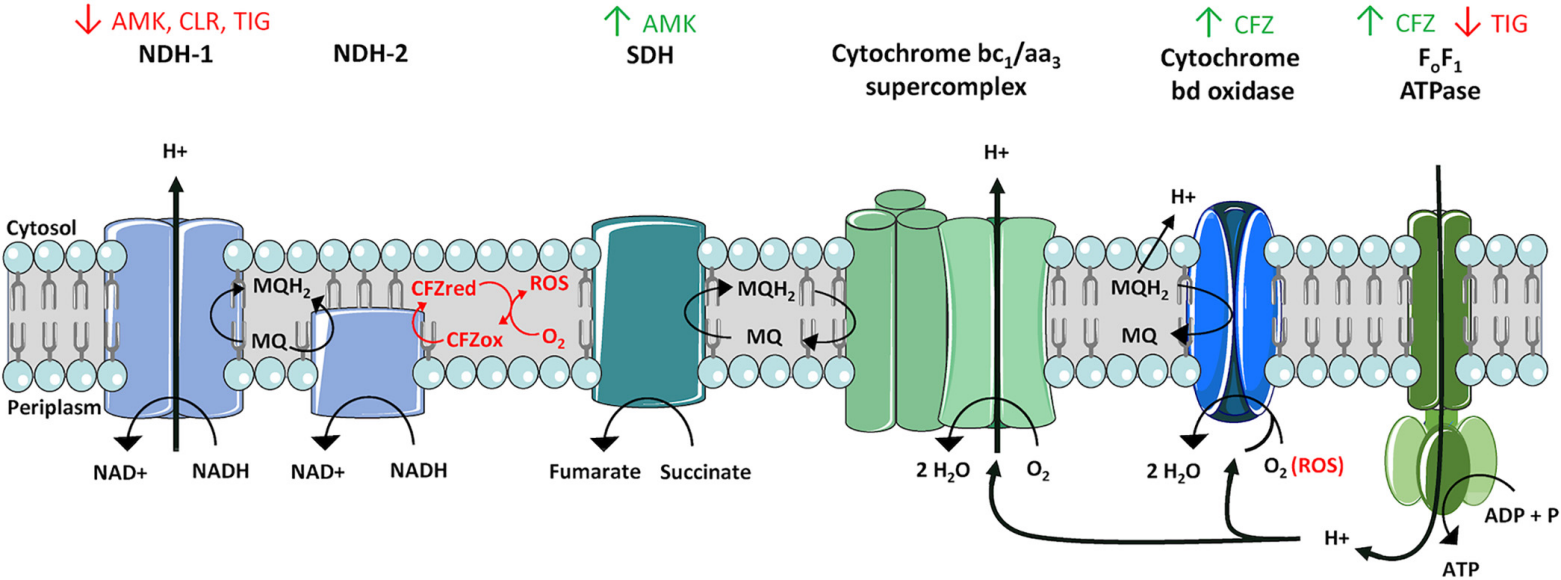
The mycobacterial respiratory chain. Graphical representation of the mycobacterial respiratory chain. NDH1, NADH dehydrogenase subtype 1; NDH2, NADH dehydrogenase subtype 2; SDH, succinate dehydrogenase. Green and red arrows indicate complexes for which encoding genes are up- or downregulated, respectively. The proposed redox cycling pathway of clofazimine is depicted in red (24).

Gene Ontology (GO) enrichment was performed on DEG sets (Fig. S2 in the supplemental material) and showed the response to ribosome-targeting drugs is characterized by both common and drug-specific pathways, shown in Fig. 1B The common response to ribosome-targeting drugs can be characterized by four transcriptomic responses: 1) induction of *WhiB7*, a mycobacterial transcriptional regulator responsible for antibiotic resistance; 2) a downregulation of the type I NADH dehydrogenase (*NuoA-N*), responsible for oxidizing NADH to NAD+ and thereby providing electrons for the electron transport chain; 3) the upregulation of genes related to ribosome structure and function and, and 4) increased transcription of genes encoding a wide range of transferases (detailed in Fig. 1C), including *eis2*, *aac(2’)* and *aph(3′)*. An overview of all changes in the respiratory chain following antibiotic exposure is shown in Fig. 2.

In addition, each drug induced a specific response. CLR exposure was characterized by a decreased expression of genes involved in central metabolic pathways, such as the pyruvate dehydrogenase complex and increased expression of genes involved in fatty acid catabolism (Fig. 1B). AMK exposure was characterized by the increased expression of the GroEL-GroES chaperonin system and TIG exposure led to decreased expression in both the F^1^F^0^ type ATPase and cyctochrome C reductase, both respiratory chain components, and increased expression of the *sigH* sigma factor (Fig. 1B).

### Cross-over experiment results.

**FIG 3:**
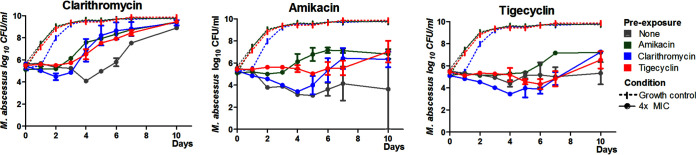
Time-kill curves following preexposure. Log-phase bacteria were exposed to subinhibitory concentrations ofamikacin (8 μg/ml), clarithromycin (4 μg/ml), or tigecycline (0.5 μg/ml) for 24 h. Subsequently, a 0.5 McFarland was made, and time-kill kinetic analysis was performed using 4× MIC of each drug.

To study the effect of the overlapping transcriptomic response to ribosomal-targeting drugs, we performed time-kill kinetic analysis using strains preexposed to each drug for 24 h. Time-kill kinetic analyses show that preexposure also influences subsequent killing of 4× MICs of each drug (Fig. 3, Table 1). The most striking effect of preexposure is seen in the more rapid emergence of macrolide resistance at day 3 in all preexposed strains due to the timely induction of *erm(41)*, leading to a decrease in effect size of between 37 and 47%. Furthermore, although less pronounced than for macrolides, the overall susceptibility to AMK is decreased, as evidenced by an absence (in TIG and AMK preexposed strains) or decrease (CLR preexposed strains) of the initial bactericidal effect of AMK in comparison to the non-exposed control. Effect sizes for AMK decreased by 29, 35, and 45% for CLR, TIG, and AMK, respectively. Finally, only preexposure to AMK led to decreased activity of TIG (23% decrease in effect size), with increased outgrowth seen from day 6.

**TABLE 1:** Effect sizes^*a*^ of time-kill kinetic curves

4× MIC	Preexposure	Effect size E (log10 CFU/ml day)
Clarithromycin	None	29,27
	Clarithromycin	15,65
	Amikacin	16,88
	Tigecycline	18,51
Amikacin	None	52,32
	Clarithromycin	36,94
	Amikacin	28,93
	Tigecycline	34,03
Tigecycline	None	40,66
	Clarithromycin	40,29
	Amikacin	31,3
	Tigecycline	39,07

*^a^*Effect size was calculated as AUC_GC_ – AUC_Treated_ for each preexposed strain relative to their own growth control.

### Knock-out of the antisigma factor (MAB_3542c).

**FIG 4:**
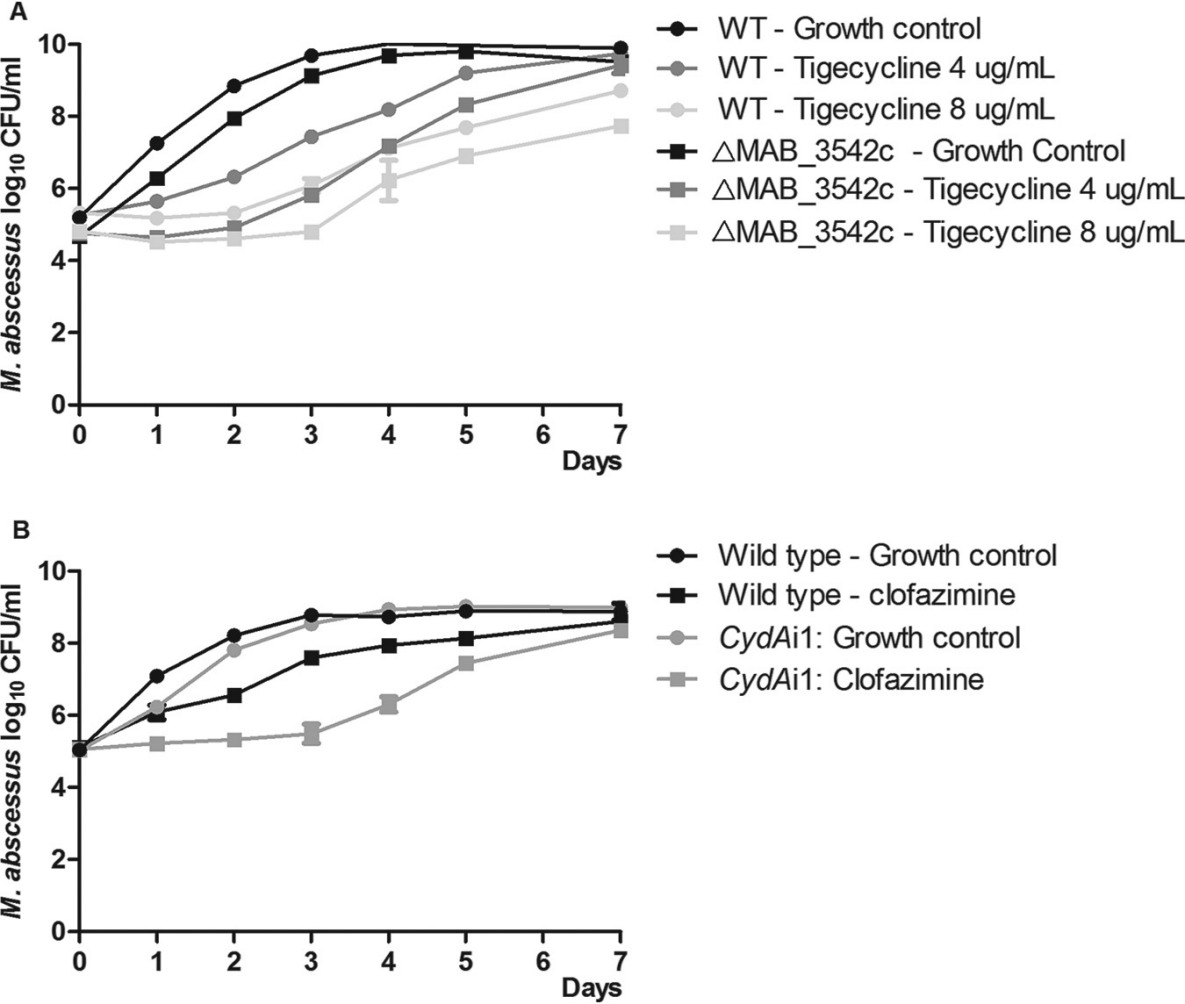
Time-kill curves of CydAi and ΔMAB_3542c. (A) Time-kill kinetic analysis was performed using 4 or 8 μg/ml tigecycline. (B) Time-kill kinetic analysis was performed using 2 μg/ml of clofazimine.

To study the role of the antisigma factor MAB_3542c on TIG resistance, we created an isogenic knockout strain, ΔMAB_3542c. Our findings show that ΔMAB_3542c only had a very minimal increase in *sigH* expression (1.19-fold increase ± SEM; 1.10–1.28); see Fig. S4A in the supplemental material. In addition, no increase in TIG MIC was seen in ΔMAB_3542c and time-kill kinetic analysis showed that the kill-curves of 1 and 2x MIC of TIG in ΔMAB_3542c and wild type were highly similar (effect sizes of 7.8 [1× MIC] and 16.5 [2× MIC] in wild type and 10.9 [1× MIC] and 19.7 [2× MIC] in ΔMAB_3542c), shown in Fig. 4A and Table 2.

**TABLE 2:** Effect sizes^*a*^ of time-kill kinetic curves for CydAi and ΔMAB_3542c

Antibiotic exposure	Strain	Effect size E (log10 CFU/ml day)
Clofazimine (2 μg/ml)	CydAi1	12,15
	Wild type	5,99
Tigecycline (4 μg/ml)	ΔMAB_3542c	10,89
	Wild type	7,84
Tigecycline (8 μg/ml)	ΔMAB_3542c	19,72
	Wild type	16,54

*^a^*Effect size was calculated as AUC_GC_ – AUC_Treated_ for each preexposed strain relative to their own growth control.

### The transcriptomic response to FOX and CFZ.

Exposure to FOX showed a relatively small amount of DEGs compared with ribosome-targeting antibiotics, only 32 (up- and downregulated) at 24 h. Only in the late response an increased expression of the *iniBAC* operon was seen. Exposure to CFZ yielded an even more limited transcriptomic response both at 4 and 24 h postexposure. The early time point showed upregulation of only two genes, a hydroperoxide reductase and a peroxiredoxin. At 24 h, CFZ exposure still only led to 14 DEGs (up- and downregulated), including structural components of the cytochrome bd-type quinol oxidase and the CydC/CydD transporter. GO enrichment showed additional increased expression of pathways related to the F^1^F^0^-ATP synthase.

### CRISPR interference of the bd-type quinol oxidase.

To confirm the role of the respiratory chain, particularly the bd-type quinol oxidase, in CFZ susceptibility we created isogenic inducible knockdown mutants of *cydA*, a subunit of the bd type quinol oxidase. All *cydA*-targeting guides were successful in increasing susceptibility to CFZ in comparison to the wild-type strain until day seven, shown in Fig. S3. Overall *cydA*i1 had the largest increase in CFZ susceptibility, accompanied by a doubling in effect size (Fig. 4B and Table 2). We then confirmed that the increased CFZ susceptibility was due to decreased *cydA* expression, with a mean fold reduction of 0,30 (fold reduction +- SEM; 0,18 – 0,52); see Fig. S4B

### Pan-genome analysis results.

To study genomic plasticity in M. abscessus we performed pan-genome analysis of all full-length M. abscessus genomes on NCBI and two novel M. abscessus genomes obtained using PacBio long-read sequencing. The M. abscessus genome contains 3,183 core genes (present in >99% of strains), 522 belonged to the soft core genes (present in 95–99% of strains), 2,160 shell genes (present in 15–95% of strains), and 5,846 cloud genes (present in <15% of strains), contributing to a total gene universe of 11,714 genes.

Subsequently, strain phylogeny and protein similarity between the M. abscessus ATCC 19977 and all included strains for all the genes indicated to be of importance for resistance were studied. Most included proteins have a high level (>95%) of homology. One exception is *erm(41)*, in which a large deletion distinguishes M. abscessus subspecies *massiliense* isolates from the other subspecies. In addition, *sigH* and, *aac2’* were each found to have multiple partial matches of the total sequence in the same region in a single strain, likely due to misassembly in the published genomes.

## DISCUSSION

Mycobacterium abscessus employs highly tailored responses to the antibiotics in currently recommended regimens. Key features of its tolerance to antibiotics are drug-specific converting enzymes, target protection and shifts in its respiratory chain and metabolic state. The mechanism of action of CFZ is still incompletely understood, but based on the transcriptomic response and CRISPR interference experiments we shed new light on the importance of the cytochrome bd-type quinol oxidase in CFZ tolerance in M. abscessus. Ribosome-targeting antibiotics elicit an elevated transcriptional response in overlapping pathways such as *WhiB7* in M. abscessus, resulting in cross-resistance between these antibiotics, as seen in time-kill analysis. The sigma factor *sigH* is thought to be important for tigeycline resistance, however, knockout of antisigma factor MAB_3542c does not influence *sigH* regulation and TIG resistance. Genes essential in all these responses are part of the core genome and show high degrees of homology, with the known exception of *erm*(41) which has a large deletion in all strains of M. abscessus subsp. *massiliense*, rendering these bacteria susceptible to macrolide antibiotics (6).

### The transcriptomic response to ribosome-targeting drugs.

The common transcriptional response to ribosome-targeting drugs consists of the induction of *WhiB7*, genes encoding drug-modifying transferases and genes involved in ribosome biogenesis (see Fig. S2 in the supplemental material), accompanied by a downregulation of respiration through the NADH dehydrogenase encoded by *Nuo(A-N)*.

We measured increased expression of *whiB7* after exposure to all ribosome targeting antibiotics. While it has previously been shown that CLR induces *whiB7*, there are conflicting findings with regard to AMK. Although *whiB7* knockout studies have shown increased susceptibility to AMK, Pryjma et al. found that AMK did not induce expression of whiB7 after 3 h of incubation (14). In line with previous findings (15), we show that AMK-induced expression of *whiB7* increases over time and is approximately 40-fold higher after 24 h than 4 h. Confirming that AMK is weaker inducer of w*hiB7* and that the induction is time-dependent.

The NADH dehydrogenase encoded by *nuo(A-N)* contributes to the proton-motive force in the electron transport chain (16). Because a proton-motive force is necessary for passive diffusion of molecules into the intracellular space, the decrease in *nuo(A-N)* expression may also contribute to increased tolerance to aminoglycosides, as their uptake has previously been shown to be dependent on proton-motive force (17).

In mycobacteria, N-acetyl and phosphotransferases convert aminoglycoside antibiotics leading to decreased susceptibility (8). Many of these transferases were upregulated following exposure to all ribosome-targeting antibiotics many of these transferases were upregulated; shown in Fig. 1C. While expression of the well-known aminoglycoside converting enzyme-encoding genes *eis2* and *aph(3′)* (8) was increased following exposure to all three antibiotics, *aac(2’)* (8), was not induced by AMK. This, together with the altered structure of AMK in comparison to other aminoglycosides likely explains why some activity of AMK in M. abscessus remains.

The expression of structural and functional components of the ribosome and increasing levels of ribosomal protein transcription is known from Escherichia coli as an attempt to recover ribosomal function (18).

### Cross-resistance following exposure to ribosome-targeting drugs.

As *whiB7* expression is induced by all ribosome-targeting antibiotics, it stands to reason that preexposure to any drug within these classes, and perhaps others, might decrease the therapeutic success of subsequent treatment with the other drugs. The time-kill assays demonstrate that preexposure to any of the ribosome-targeting antibiotics leads to more rapid emergence of macrolide resistance and decreased susceptibility to AMK and TIG (Fig. 3), most likely due to the shared upregulation of w*hiB7*. The strongest effect is seen in the emergence of macrolide resistance at day 3 due to the timely induction of *erm(41)*. In addition, *WhiB7* mediated induction of aminoglycoside-modifying enzymes at an early time point does likely contribute to increased AMK tolerance. However, this is less clear in CLR preexposed samples that initially show increased killing, possibly due to their altered metabolism. Lastly, while the effect on growth in the presence of TIG is less pronounced, overall, the drug susceptibility is decreased. This is of particular concern in patients receiving macrolide or aminoglycoside maintenance therapy while at high-risk for M. abscessus infection, such as CF and bronchiectasis patients. This low-level antibiotic exposure may contribute to decreased therapeutic success of subsequent M. abscessus treatment. Our findings highlight the importance of screening patients for NTM prior to considering maintenance therapy (12).

### CLR exposure induces a persister-like state.

CLR exposure induces a downregulation of the type I NADH dehydrogenase [Nuo(A-N)] and a decreased expression of the pyruvate dehydrogenase complex, the main link between glycolysis and the TCA cycle. This was accompanied by an increased expression of genes involved in fatty acid catabolism at 24 h. This decreased respiration through Nuo(A-N) and fatty acid catabolism to provide acetyl-coA for the TCA cycle through β-oxidation is similar to the shifts seen in M. tuberculosis in its non-replicating persister state, associated with decreased drug susceptibility (19, 20). Our findings indicate that M. abscessus, upon macrolide exposure, decreases flux through the TCA cycle, thereby inducing tolerance, while fatty acids are utilized to maintain low-level metabolic activity until resistance is induced via the *erm(41)* gene (6).

### Aminoglycoside tolerance due to chaperonin-guided protein folding.

Following 24 h of AMK exposure upregulation of chaperonin proteins GroEL and GroES was seen. Aminoglycoside antibiotics function by interrupting protein translation and inhibiting proper protein folding (21). The GroEL-GroES chaperonin system is known to assist in folding and has been shown to protect E. coli from aminoglycoside activity (22). This is also likely the case for M. abscessus.

### TIG.

We observed an increased expression of sigma factor H (*sigH*) following TIG exposure. Recently, Ng et al. showed that a point mutation in the antisigma factor MAB_3542c in M. abscessus, the inhibitor of *sigH* (MAB_3543c), leads to increased TIG resistance, likely by decreased inhibition of *sigH* (23). Therefore, increased expression of *sigH* may contribute to increased TIG tolerance. To study the role of MAB_3542c on TIG resistance further we constructed a knockout strain, ΔMAB_3542c. The MIC of TIG in ΔMAB_3542c was not increased in comparison to the wild type (WT) and time-kill kinetic curves only showed a small decrease in susceptibility that is more likely explained by an inoculum effect and biological variation in response (fig. 4B). In addition, the expression of *sigH* in ΔMAB_3542c was also not increased in comparison to WT (Fig. S3B). These findings contrast with previous findings by Ng et al. (23), that suggest MAB_3542c is an inhibitor of *sigH* transcription and that decreased inhibition leads to TIG resistance. No increased expression of the *MabTetX* (MAB_1496c) tetracycline monooxygenase was observed; this confirms the previous finding that TIG is a poor substrate -and does not induce expression- of the gene encoding *MabTetX* (9).

### FOX and CFZ induce small specific transcriptomic responses.

FOX exposure only led to increased expression of genes involved in DNA-directed RNA polymerase activity. Interestingly, following FOX exposure no increase in transcription of *Bla_Mab* was seen, although M. abscessus bacteria were able to grow at only a slightly decreased rate compared to the growth controls. Constitutive expression of the β-lactamase gene is likely sufficient for protection against the relatively low level of β-lactam exposure.

CFZ exposure led to an increased expression of genes encoding components of the respiratory chain. Upregulation included structural components of the F^1^F^0^-ATP synthase, the cytochrome bd-type quinol oxidase (CydA and CydB) and the CydC/CydD transporter thought to be essential for the function of the cytochrome bd-type ubiquinol oxidoreductase in M. tuberculosis (19). Although the exact mechanisms of action of CFZ is not fully understood, our findings suggest that increased oxidative stress contributes to the bactericidal effect, supporting the previously proposed redox cycling pathway of CFZ, in which Reactive Oxygen Species are generated (24). Upregulation of the cytochrome bd-type quinol oxidase likely helps to tolerate CFZ, as it has previously been shown to protect M. smegmatis from oxidative stress while depletion of the oxidase lead to increased susceptibility to CFZ (25). This theory is further supported by our findings using isogenic inducible knockdown of *cydA* in M. abscessus. When transcription of *cydA* was limited an increase in susceptibility to CFZ was seen (Fig. 4A), thereby confirming the role of the cytochrome bd-type quinol oxidase in CFZ tolerance in M. abscessus. As the cytochrome bd-type quinol oxidase is only found in prokaryotes this is a promising drug-target, potentially in combination with CFZ, for this extremely difficult-to-treat pathogen (26).

Here we provide the first description of the transcriptomic antibiotic stress response of M. abscessus over the breadth of all drugs included in treatment regimens. However, while this unbiased method allows identification of overlapping and drug-specific responses, it is in many instances hypothesis-generating in nature and still requires in-depth analyses into all discovered pathways. We believe that future work characterizing the proposed mechanisms would be of great value as transcriptomic changes do not always adequately reflect biological meaningfulness (27, 28).

In conclusion, Mycobacterium abscessus shows both drug-specific and common transcriptomic responses to antibiotic exposure. Key features of its tolerance to antibiotic exposure are drug-specific converting enzymes, target protection, and shifts in its respiratory chain and metabolic state. In confirmatory experiments, we highlight the role of the bd-type quinol oxidase in CFZ tolerance, suggesting ROS generation is a key mechanisms of action of CFZ, and role of the SigH sigma factor but not the MAB_3542c antisigma factor in TIG tolerance. Because of the common response elicited by ribosome-targeting antibiotics, exposure to any of these drugs rapidly induces tolerance mechanisms that decrease susceptibility to ribosome-targeting drugs from multiple classes. A deeper understanding of drug tolerance and resistance is essential to improve antibiotic treatment regimens and their outcome.

## MATERIALS AND METHODS

### Bacterial strains and antibiotics.

The M. abscessus subsp. *abscessus* ATCC 19977 type strain (Institute Pasteur, Paris, France) with established MICs of 32, 4, 4, 64 and 0.5 μg/ml for AMK, CLR, TIG, FOX, and CFZ was used. Stock vials of the strain were stored at −70°C in Trypticase soy broth containing 40% glycerol. CLR, AMK, FOX, and CFZ were obtained from Sigma-Aldrich (Zwijndrecht, The Netherlands) and TIG was obtained from Pfizer (Capelle aan den Ijssel, The Netherlands). Antibiotics were dissolved in methanol, MilliQ, MilliQ, DMSO and, MilliQ, respectively.

### Culture conditions.

A bacterial inoculum was grown before each assay, diluted in Cation Adjusted Mueller-Hinton (CAMH; for CLR, AMK, TIG, and FOX conditions) or Middlebrook 7H9 (for CFZ conditions) broth with 0.05% Tween 80 and cultured until early log-phase, see supplementary methods for more detailed protocols. Subsequently, antibiotics at subinhibitory concentrations (CLR [4 μg/ml], AMK [8 μg/ml], TIG [0,5 μg/ml], FOX [8 μg/ml], or CFZ [1 μg/ml]) were added and the culture was grown for a further 4 or 24 h prior to RNA isolation. All conditions were completed in biological triplicate.

### RNA isolation and library preparation.

RNA isolation was performed using the Nucleospin RNA kit (Machery Nagel, Düren, Germany), with slight modifications, a detailed protocol is included in supplementary methods. Following isolation, RNA integrity was determined, rRNA was depleted and the mRNA library was constructed and sequenced on a NextSeq 500 (Illumina, San Diego, CA).

### Differential gene expression analysis.

All obtained reads were mapped to the M. abscessus ATCC 17799 genome using STAR (v2.7.0) (29). Differential expression analysis was performed in R (v3.3.3) using the DESeq2 package (30) and cutoffs were defined as Log_2_ fold change ≥ 2 or ≤ −2 and a *P value* corrected for multiple guessing ≤ 0.05. Subsequently all Gene Ontology (GO) terms were retrieved from MycoBASE (31), and an enrichment analysis was performed using the topGO R package.

### Time-kill kinetic analysis following 24-h preexposure of ribosome-targeting drugs.

Duplicate vials containing CAMH with 0.05% Tween 80 and either 4× MIC of CLR, AMK, or TIG were inoculated using M. abscessus preexposed to one of these antibiotics for 24 h, thereby mimicking the exposure in transcriptomic experiments, alongside an antibiotic-free growth control. Vials were incubated on shaker at 100 rpm at 30°C with oxygenation through a filtered needle. Growth of the bacterial population over 8 days of exposure was quantified by CFU counting using a 10-fold serial dilution on Middlebrook 7H10 agar (BD Bioscience, Erembodegem, Belgium) and incubated at 30°C for 3 days before CFU counting. The AUC was calculated from log CFU over time using the trapezoidal rule after averaging the results from both replicates in GraphPad prism version 5.03. Effect size was then calculated as AUC_GC_ –AUC_Treated_ for each preexposed strain relative to their own growth control, >10% decrease in effect size was considered a decreased response to treatment (32).

### Generation of isogenic inducible knockdown strains of *cydA* using dCas9.

We used the recently described inducible CRISPR interference system in M. abscessus ATCC 19977 (3). In short, the M. abscessus ATCC 19977 type strain was transformed with a dCas9 encoding plasmid (pTetInt-dcas9-Hyg) and a second vector (pGRNAz) containing a custom-designed small guide RNA (sgRNA) cassette. Four 20-nucleotide guides were designed for cydA and annealed and cloned between sphI and aclI of the pGRNAz and the respective strains were named *cydA*i1-4. In addition, a strain with nontargeting guides was included. To select for successful clones, the transformed bacteria were plated on Middlebrook 7H10 agar containing 1 mg/ml hygromycin and 300 mg/ml zeocin, and colonies were picked and stored at −70°C in Trypticase soy broth containing 40% glycerol.

### Generation of ΔMAB_3542c using mycobacterial recombineering.

We used a previously described mycobacterial recombineering system optimized for M. abscessus ATCC 19977 (33). In short, a M. abscessus recombineering strain containing the pJV53 plasmid encoding the Che9c recombination proteins was prepared. Subsequently, a custom plasmid was designed using pUc19 in which regions of homology flanking the target gene MAB_3542c were cloned in combination with a zeocin resistance cassette and transformed into the M. abscessus recombineering strain. The transformed strains were incubated in 7H9 broth containing ADC and 0,05% Tween to recover and then plated on zeocin-containing 7H10 agar to select for resistant transformants. Whole genome sequencing was performed to confirm recombinant strain genotype.

### Time-kill kinetic analysis of isogenic knockdown strains.

Strains containing dCas9 were grown in Middlebrook 7H9 broth with 0.05% Tween 80, 1 mg/ml hygromycin and 300 mg/ml zeocin and the M. abscessus WT was cultured in antibiotic-free medium. After 4 days, duplicate vials containing 10 ml Middlebrook 7H9 broth containing no CFZ or CFZ at a concentration of 8 μg/ml were inoculated as previously described. To induce gene silencing anhydrotetracycline was added at 100 ng/ml to all conditions, including the WT control, and vials were incubated as described previously. Growth of bacterial populations was followed for 7 days on Mueller-Hinton II agar and effect size was calculated as previously described.

### MIC and Time-kill kinetic analysis of isogenic knockout strains.

ΔMAB_3542c was grown in Middlebrook 7H9 broth with 0.05% Tween 80 and 300 mg/ml zeocin and the M. abscessus WT was cultured in antibiotic-free medium for 4 days. TIG MIC was determined using sensititre RAP plates following CLSI guidelines. For time-kill kinetic analysis, duplicate vials containing 10 ml CAMH broth containing no TIG or TIG at a concentration of either 4 or 8 μg/ml were inoculated and growth of bacterial populations was followed for 7 days on Middlebrook 7H11 II agar and effect size was calculated as previously described.

### qPCR confirmation of *cydA* and *sigH* expression.

*CydA*i1 and ΔMAB_3542c strains were cultured as previously described in the absence of CFZ or TIG. The WT M. abscessus was included as a control for the ΔMAB_3542c strain and a strain containing pGRNAz with non-targeting guides was included as a control for *CydA*i1. Three biological replicates of each condition were included. The relative expression of cydA and sigH was determined in comparison to the housekeeping genes Glyceraldehyde 3-phosphate dehydrogenase and RNA polymerase subunit beta. Primer sequences and PCR protocol are shown in Tables S1 and S2 in the supplemental material.

### gDNA isolation and PacBio sequencing.

Genomic DNA (gDNA) was isolated using the Bacterial gDNA isolation kit (Norgen Biotek Corp, Thorold, Canada) with slight modifications, see supplementary methods for detailed protocol. All gDNA fragments above 4 kb were selected using the BluePippin (Sage Science, Beverly, MA), samples were prepared for PacBio SMRT and sequenced on a Sequel SMRT Cell 1M v2 (Pacific Biosciences, Menlo Park, CA).

### *De novo* long-read assembly and phylogeny analysis.

*De novo* genome assembly was performed using Canu (v1.9) and annotated with PROKKA (v1.14.5). Phylogeny analysis of included strains was performed using RAxML (v8.0.0) and the tree was rooted at midpoint. Subsequently, ROARY (v3.13.0) was used to determine the core and accessory genome, genes were divided into core (present in >99% of strains), soft core (present in 95–99% of strains), shell (present in 15–95% of strains), and cloud (present in <15% of strains) (34). Finally, the presence and protein sequence homology of genes was determined using BLAST (v2.9.0+).

### Data availability.

Raw PacBio data are publicly available in the SRA database under BioProject number PRJNA655109. Raw illumina read data has been uploaded to the Gene Expression Omnibus database under accession no. GSE163621.

## References

[1] Schuurbiers MMF, Bruno M, Zweijpfenning SMH, Magis-Escurra C, Boeree M, Netea MG, van Ingen J, van de Veerdonk F, Hoefsloot W. 2020. Immune defects in patients with pulmonary Mycobacterium abscessus disease without cystic fibrosis. ERJ Open Res 6:00590-2020. 10.1183/23120541.00590-2020. 33263065 PMC7682720

[2] Bryant JM, Grogono DM, Rodriguez-Rincon D, Everall I, Brown KP, Moreno P, Verma D, Hill E, Drijkoningen J, Gilligan P, Esther CR, Noone PG, Giddings O, Bell SC, Thomson R, Wainwright CE, Coulter C, Pandey S, Wood ME, Stockwell RE, Ramsay KA, Sherrard LJ, Kidd TJ, Jabbour N, Johnson GR, Knibbs LD, Morawska L, Sly PD, Jones A, Bilton D, Laurenson I, Ruddy M, Bourke S, Bowler IC, Chapman SJ, Clayton A, Cullen M, Daniels T, Dempsey O, Denton M, Desai M, Drew RJ, Edenborough F, Evans J, Folb J, Humphrey H, Isalska B, Jensen-Fangel S, Jönsson B, Jones AM, et al. 2016. Emergence and spread of a human-transmissible multidrug-resistant nontuberculous mycobacterium. Science 354:751–757. 10.1126/science.aaf8156. 27846606 PMC5142603

[3] Bryant JM, Brown KP, Burbaud S, Everall I, Belardinelli JM, Rodriguez-Rincon D, Grogono DM, Peterson CM, Verma D, Evans IE, Ruis C, Weimann A, Arora D, Malhotra S, Bannerman B, Passemar C, Templeton K, MacGregor G, Jiwa K, Fisher AJ, Blundell TL, Ordway DJ, Jackson M, Parkhill J, Floto RA. 2021. Stepwise pathogenic evolution of Mycobacterium abscessus. Science 372:eabb8699. 10.1126/science.abb8699. 33926925 PMC7611193

[4] Nessar R, Cambau E, Reyrat JM, Murray A, Gicquel B. 2012. Mycobacterium abscessus: a new antibiotic nightmare. J Antimicrob Chemother 67:810–818. 10.1093/jac/dkr578. 22290346

[5] Chiaradia L, Lefebvre C, Parra J, Marcoux J, Burlet-Schiltz O, Etienne G, Tropis M, Daffé M. 2017. Dissecting the mycobacterial cell envelope and defining the composition of the native mycomembrane. Sci Rep 7:12807. 10.1038/s41598-017-12718-4. 28993692 PMC5634507

[6] Nash KA, Brown-Elliott BA, and Wallace RJ, Jr, Novel A. 2009. gene, erm(41), confers inducible macrolide resistance to clinical isolates of Mycobacterium abscessus but is absent from Mycobacterium chelonae. Antimicrob Agents Chemother 53:1367–1376. 10.1128/AAC.01275-08. 19171799 PMC2663066

[7] Rominski A, Selchow P, Becker K, Brülle JK, Dal Molin M, Sander P. 2017. Elucidation of Mycobacterium abscessus aminoglycoside and capreomycin resistance by targeted deletion of three putative resistance genes. J Antimicrob Chemother 72:2191–2200. 10.1093/jac/dkx125. 28486671

[8] Luthra S, Rominski A, Sander P. 2018. The role of antibiotic-target-modifying and antibiotic-modifying enzymes in Mycobacterium abscessus drug resistance. Front Microbiol 9:2179. 10.3389/fmicb.2018.02179. 30258428 PMC6143652

[9] Rudra P, Hurst-Hess K, Lappierre P, Ghosh P. 2018. High levels of intrinsic tetracycline resistance in Mycobacterium abscessus are conferred by a tetracycline-modifying monooxygenase. Antimicrob Agents Chemother 62. 10.1128/AAC.00119-18. PMC5971581 29632012

[10] Richard M, Gutiérrez AV, Viljoen A, Rodriguez-Rincon D, Roquet-Baneres F, Blaise M, Everall I, Parkhill J, Floto RA, Kremer L. 2019. Mutations in the MAB_2299c TetR regulator confer cross-resistance to clofazimine and bedaquiline in Mycobacterium abscessus. Antimicrob Agents Chemother 63. 10.1128/AAC.01316-18. PMC6325171 30323043

[11] Daley CL, Iaccarino JM, Lange C, Cambau E, Wallace RJ, Andrejak C, Böttger EC, Brozek J, Griffith DE, Guglielmetti L, Huitt GA, Knight SL, Leitman P, Marras TK, Olivier KN, Santin M, Stout JE, Tortoli E, van Ingen J, Wagner D, Winthrop KL. 2020. Treatment of nontuberculous Mycobacterial pulmonary disease: An official ATS/ERS/ESCMID/IDSA clinical practice guideline. Clin Infect Dis 71:905–913. 10.1093/cid/ciaa1125. 32797222 PMC7768745

[12] Floto RA, Olivier KN, Saiman L, Daley CL, Herrmann J-L, Nick JA, Noone PG, Bilton D, Corris P, Gibson RL, Hempstead SE, Koetz K, Sabadosa KA, Sermet-Gaudelus I, Smyth AR, van Ingen J, Wallace RJ, Winthrop KL, Marshall BC, Haworth CS, US Cystic Fibrosis Foundation and European Cystic Fibrosis Society. 2016. US Cystic Fibrosis Foundation and European Cystic Fibrosis Society consensus recommendations for the management of non-tuberculous mycobacteria in individuals with cystic fibrosis. Thorax 71 Suppl 1:i1–22. 10.1136/thoraxjnl-2015-207360. 26666259 PMC4717371

[13] Kwak N, Dalcolmo MP, Daley CL, Eather G, Gayoso R, Hasegawa N, Jhun BW, Koh W-J, Namkoong H, Park J, Thomson R, van Ingen J, Zweijpfenning SMH, Yim J-J. 2019. Mycobacterium abscessus pulmonary disease: individual patient data meta-analysis. Eur Respir J 54:1801991. 10.1183/13993003.01991-2018. 30880280

[14] Pryjma M, Burian J, Kuchinski K, Thompson CJ. 2017. Antagonism between front-line antibiotics clarithromycin and amikacin in the treatment of Mycobacterium abscessus infections is mediated by the whiB7 gene. Antimicrob Agents Chemother 61. 10.1128/AAC.01353-17. PMC5655113 28874379

[15] Hurst-Hess K, Rudra P, and, Ghosh P. 2017. Mycobacterium abscessus WhiB7 regulates a species-specific repertoire of genes to confer extreme antibiotic resistance. Antimicrob Agents Chemother 61. 10.1128/AAC.01347-17. PMC5655061 28874378

[16] Cook GM, Hards K, Vilchèze C, Hartman T, Berney M. 2014. Energetics of respiration and oxidative phosphorylation in mycobacteria. Microbiol Spectr 2. 10.1128/microbiolspec.MGM2-0015-2013. PMC4205543 25346874

[17] Raaijmakers J, et al. 2021. The role of amikacin in the treatment of nontuberculous mycobacterial disease. Expert Opin Pharmacother 22:1961–1974. 10.1080/14656566.2021.1953472. 34292097

[18] Dodd J, Kolb JM, Nomura M. 1991. Lack of complete cooperativity of ribosome assembly in vitro and its possible relevance to in vivo ribosome assembly and the regulation of ribosomal gene expression. Biochimie 73:757–767. 10.1016/0300-9084(91)90055-6. 1764521

[19] Shi L, Sohaskey CD, Kana BD, Dawes S, North RJ, Mizrahi V, Gennaro ML. 2005. Changes in energy metabolism of Mycobacterium tuberculosis in mouse lung and under in vitro conditions affecting aerobic respiration. Proc Natl Acad Sci USA 102:15629–15634. 10.1073/pnas.0507850102. 16227431 PMC1255738

[20] Maurya RK, Bharti S, and, Krishnan MY. 2018. Triacylglycerols: Fuelling the Hibernating Mycobacterium tuberculosis. Front Cell Infect Microbiol 8:450. 30687647 10.3389/fcimb.2018.00450 PMC6333902

[21] Goltermann L, Good L, and, Bentin T. 2013. Chaperonins fight aminoglycoside-induced protein misfolding and promote short-term tolerance in Escherichia coli. J Biol Chem 288:10483–10489. 10.1074/jbc.M112.420380. 23447537 PMC3624430

[22] Goltermann L, Sarusie MV, and, Bentin T. 2015. Chaperonin GroEL/GroES over-expression promotes aminoglycoside resistance and reduces drug susceptibilities in Escherichia coli following exposure to sublethal aminoglycoside doses. Front Microbiol 6:1572. 26858694 10.3389/fmicb.2015.01572 PMC4726795

[23] Ng HF, Tan JL, Zin T, Yap SF, Ngeow YF. 2018. A mutation in anti-sigma factor MAB_3542c may be responsible for tigecycline resistance in Mycobacterium abscessus. J Med Microbiol 67:1676–1681. 10.1099/jmm.0.000857. 30351265

[24] Yano T, Kassovska-Bratinova S, Teh JS, Winkler J, Sullivan K, Isaacs A, Schechter NM, Rubin H. 2011. Reduction of clofazimine by mycobacterial type 2 NADH:quinone oxidoreductase: a pathway for the generation of bactericidal levels of reactive oxygen species. J Biol Chem 286:10276–10287. 10.1074/jbc.M110.200501. 21193400 PMC3060482

[25] Lu P, Heineke MH, Koul A, Andries K, Cook GM, Lill H, van Spanning R, Bald D. 2015. The cytochrome bd-type quinol oxidase is important for survival of Mycobacterium smegmatis under peroxide and antibiotic-induced stress. Sci Rep 5:10333. 10.1038/srep10333. 26015371 PMC4450806

[26] Beites T, O'Brien K, Tiwari D, Engelhart CA, Walters S, Andrews J, Yang H-J, Sutphen ML, Weiner DM, Dayao EK, Zimmerman M, Prideaux B, Desai PV, Masquelin T, Via LE, Dartois V, Boshoff HI, Barry CE, Ehrt S, Schnappinger D. 2019. Plasticity of the Mycobacterium tuberculosis respiratory chain and its impact on tuberculosis drug development. Nat Commun 10:4970. 10.1038/s41467-019-12956-2. 31672993 PMC6823465

[27] Vogel C, and, Marcotte EM. 2012. Insights into the regulation of protein abundance from proteomic and transcriptomic analyses. Nat Rev Genet 13:227–232. 10.1038/nrg3185. 22411467 PMC3654667

[28] Koussounadis A, Langdon SP, Um IH, Harrison DJ, Smith VA. 2015. Relationship between differentially expressed mRNA and mRNA-protein correlations in a xenograft model system. Sci Rep 5:10775. 10.1038/srep10775. 26053859 PMC4459080

[29] Dobin A, Davis CA, Schlesinger F, Drenkow J, Zaleski C, Jha S, Batut P, Chaisson M, Gingeras TR. 2013. STAR: ultrafast universal RNA-seq aligner. Bioinformatics 29:15–21. 10.1093/bioinformatics/bts635. 23104886 PMC3530905

[30] Love MI, Huber W, and, Anders S. 2014. Moderated estimation of fold change and dispersion for RNA-seq data with DESeq2. Genome Biol 15:550. 10.1186/s13059-014-0550-8. 25516281 PMC4302049

[31] Garcia BJ, Datta G, Davidson RM, Strong M. 2015. MycoBASE: expanding the functional annotation coverage of mycobacterial genomes. BMC Genomics 16:1102. 10.1186/s12864-015-2311-9. 26704706 PMC4690229

[32] Sonawane VV, Ruth MM, Pennings LJ, Svensson EM, Wertheim HFL, Hoefsloot W, van Ingen J. 2021. An in vitro perspective on what individual antimicrobials add to Mycobacterium avium complex therapies. Antimicrob Agents Chemother 65:e0273020. 10.1128/AAC.02730-20. 33972258 PMC8284439

[33] van Kessel JC, and, Hatfull GF. 2008. Mycobacterial recombineering. Methods Mol Biol 435:203–215. 10.1007/978-1-59745-232-8_15. 18370078

[34] Page AJ, Cummins CA, Hunt M, Wong VK, Reuter S, Holden MTG, Fookes M, Falush D, Keane JA, Parkhill J. 2015. Roary: rapid large-scale prokaryote pan genome analysis. Bioinformatics 31:3691–3693. 10.1093/bioinformatics/btv421. 26198102 PMC4817141

