# Supplementary material for: RNA Sequencing Elucidates Drug-Specific Mechanisms of Antibiotic Tolerance and Resistance in Mycobacterium abscessus
Source: Antimicrob Agents Chemother. 2022 Jan 18;66(1):e01509-21. doi: 10.1128/AAC.01509-21 (PMC8765290; doi:10.1128/AAC.01509-21)
Supplement: Supplemental file 1 — Supplemental material. Download AAC.01509-21-s0001.pdf, PDF file, 0.4 MB [file aac.01509-21-s0001.pdf]

Supplement

Supplementary tables

Gene target	Primer sequence
GAPDH forward	GTGACTGTCCGGGTAGGC
GAPDH reverse	AGGATGGAGTCGAACTTCAGC
rpoB forward	TACATCCTGAAGCTGCACCA
rpoB reverse	GATCGCCTCGTAGACCTTCA
cydA forward	GGCAAGTTGATGTTCCAGCA
cydA reverse	CTTTTGCAGGTCCTCTACGC
sigH forward	TGCAAGAGACGATGGTCAAG
sigH reverse	GTGATTTCCTCGGTCGGATA

Supplementary table 1 – Included primer sequences

Step	Target (°C)	(hh:mm:ss)	Cycles
Pre-incubation	95	0:05:00	1
Amplification	95	0:00:10	40
	60	0:00:20	
	72	0:00:20	

Supplementary table 2 – PCR protocol

Supplementary figures

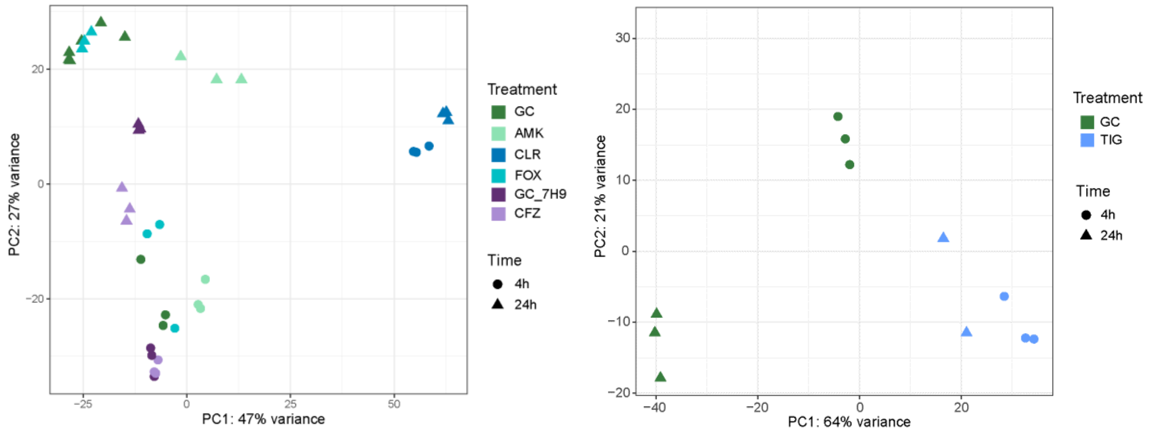

Supplementary figure 1 - PCA plot of the transcriptomic responses to all drugs included in the recommended treatment regimen, and clofazimine. A. *PCA plot of amikacin (AMK), clarithromycin (CLR), cefoxitin (FOX), clofazimine treated samples and their respective growth controls (GC & GC_7H9, respectively).* ***B.*** *PCA plot of Tigecycline (TIG) treated samples and the growth control (GC).*

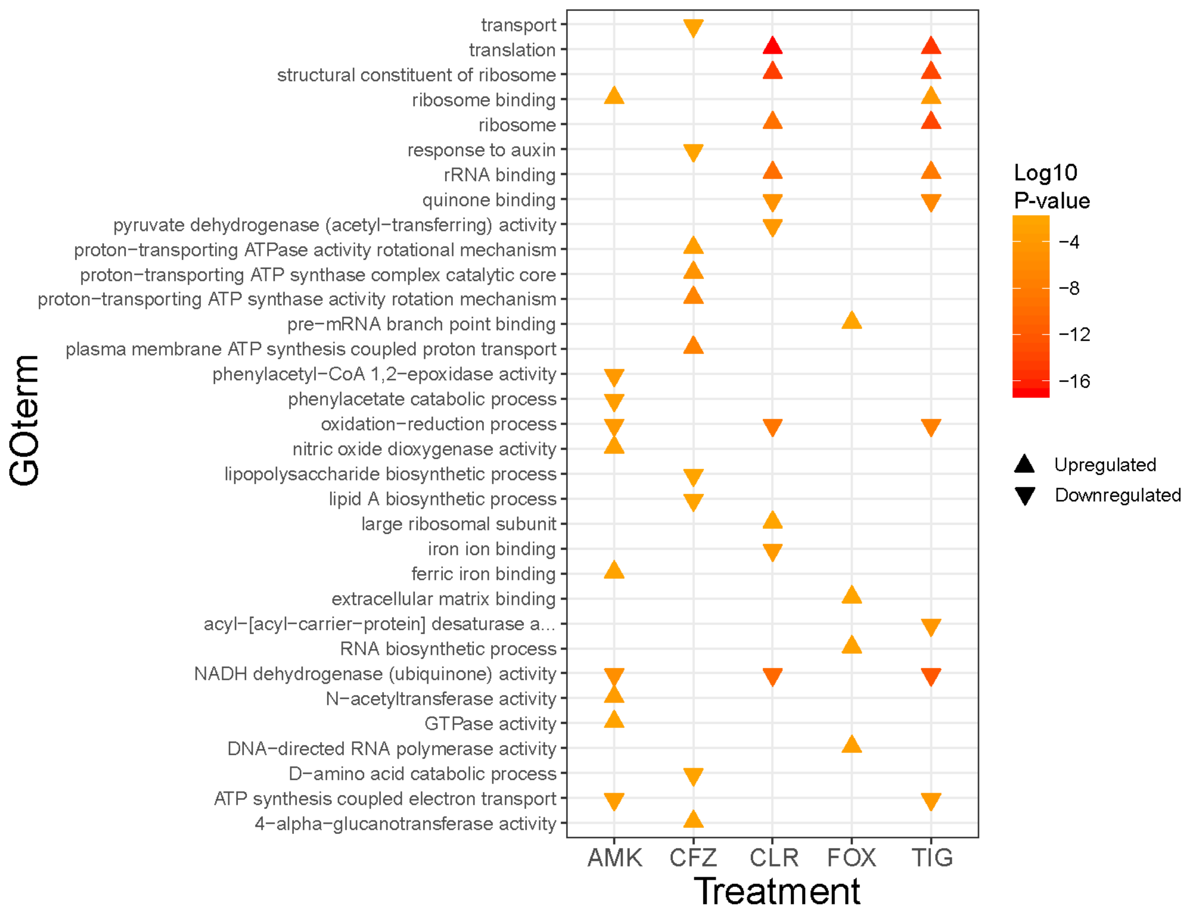

Supplementary figure 2 - GOterm enrichment. *GO enrichment was performed using the topGO package. The top 5 enriched GO terms (both up- and downregulated) from each condition are included. AMK = amikacin, CFZ = clofazimine, CLR = clarithromycin, FOX = cefoxitin, and TIG = tigecycline.*

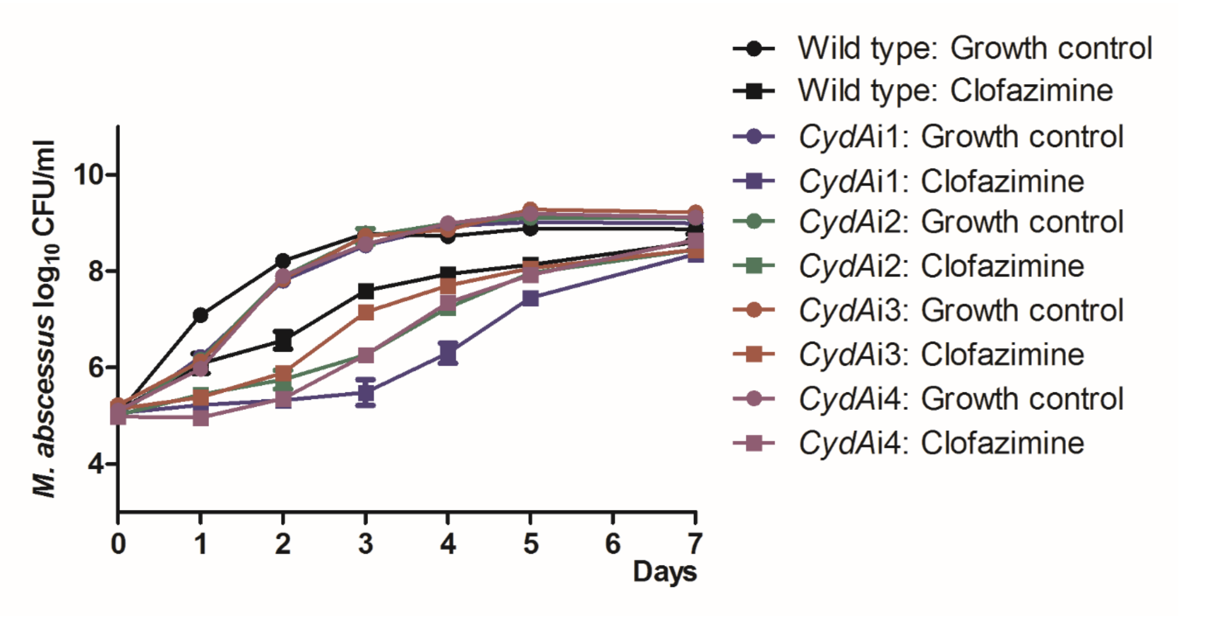

Supplementary figure 3 – Time-kill curves of CydAi1-4. *Time-kill kinetic analysis was performed using 2 µg/mL of clofazimine.*

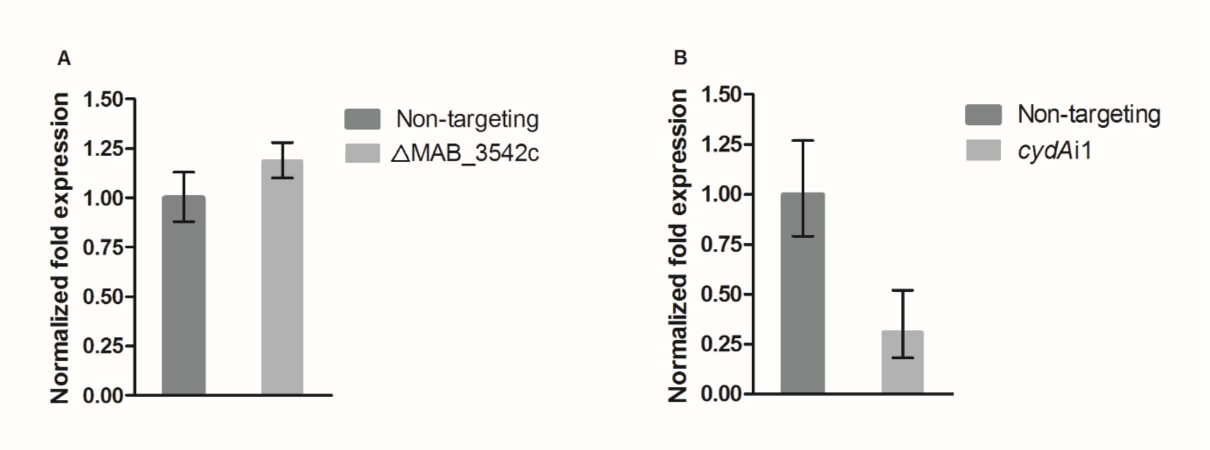

Supplementary figure 4 – Normalized fold expression of cydAi1 and ΔMAB_3542c. A*. The normalized fold expression of sigH in cydAi1 in and ΔMAB_3542c in comparison to the wild type.* ***B.*** *The normalized fold expression of cydA after induction of the dCas9 system in cydAi1 in comparison to a strain containing non-targeting guides.*

Supplementary Methods

Culture conditions prior to antibiotic exposure

A bacterial inoculum was prepared freshly before each assay by allowing them to grow for 72 hours in Middlebrook 7H9 broth containing 0.05% Tween 80 (Sigma-Aldrich, Zwijndrecht, The Netherlands) before making a 0.5 McFarland suspension. The 0.5 McFarland suspension was then diluted 100-fold in Cation Adjusted Mueller-Hinton (CAMH) broth with 0.05% Tween 80 and cultured until early log-phase (24 hours). Three replicates were included per condition, with the exception of the growth control for clarithromycin, amikacin and cefoxitin at 24-hours, of which 6 were included.

RNA isolation and library preparation

RNA isolation was performed using the Nucleospin RNA kit (Machery Nagel, Düren, Germany). The bacterial culture was collected, spun down at maximum speed for 5 minutes and resuspended in 350 ul buffer RAW with 10ul DTT. To each sample one scoop of acid-wash glass beads <106uM (Sigma-Aldrich, Saint Louis, USA) was added and samples were flash-frozen in liquid nitrogen and placed in a MagNA Lyser (Roche, Woerden, The Netherlands) bead-beater at maximum speed for 20s. Flash freezing followed by 20s in the MagNA Lyser was repeated 3 times in total. RNA integrity was then measured on a TapeStation 2200 (Agilent, Santa Clara, USA). To remove unwanted ribosomal RNA (rRNA) depletion was performed using either RiboZero Bacteria (Illumina, San Diego, US) for clarithromycin, amikacin, cefoxitin and, clofazimine samples and controls or RiboMinus (Thermo fisher, Waltham, USA) for tigecycline samples and controls followed by ethanol precipitation following manufacturers protocol. The mRNA library was then constructed using the TruSeq RNA sample preparation V2 kit (Illumina, San Diego, USA) starting from RNA fragmentation. In short, RNA was chemically fragmented prior to cDNA synthesis. End-repair was then performed on constructed cDNA, followed by A-tailing, adaptor ligation and 15 cycles of qPCR. A clean-up of the DNA using AMPure beads was performed between each step. A 1ul aliquot of the library was again run on a TapeStation 2200 to ensure all libraries had the correct length (approximately 280bp). Libraries were pooled equimolar to a final concentration of 4nM. 1.25pM of sample was sequenced in paired-end 2x 75 bp mode on a NextSeq 500 (Illumina, San Diego, US) at the Genome Technology Center of the

Radboud University Medical Center. One 24-hour tigecycline exposed sample was removed due to incomplete rRNA removal.

gDNA isolation and PacBio sequencing

PacBio sequencing of two clinical strains was performed. In short, high molecular weight gDNA was isolated using the Bacterial gDNA Isolation Kit (Norgen Biotek Corp, Thorold, Canada) kit following 2 rounds of bead beating in a MagNAlyser at 7000 rpm with cooling in liquid nitrogen between rounds. Subsequent size selection of all gDNA fragments above 4kb was performed using a 0,75% agarose cassette on the BluePippin (Sage Science, Beverly, USA) with marker S1. Mean gDNA length was determined using a gDNA tape on the TapeStation 2200 (Agilent, Santa Clara, US) and samples were prepped for PacBio SMRT sequencing using the SMRTbell Barcoded Adapter Prep kit, Barcoded Adapter Plate -96 en SMRTbell and Damage Repair kit and sequenced on a Sequel SMRT Cell 1M v2 (Pacific Biosciences, Menlo Park, US) at the Genome Technology Center of the Radboud University Medical Center.
